# Blocked *O*-GlcNAc cycling alters mitochondrial morphology, function, and mass

**DOI:** 10.1038/s41598-021-01512-y

**Published:** 2021-11-11

**Authors:** Elizabeth O. Akinbiyi, Lara K. Abramowitz, Brianna L. Bauer, Maria S. K. Stoll, Charles L. Hoppel, Chao-Pin Hsiao, John A. Hanover, Jason A. Mears

**Affiliations:** 1 Department of Pathology, Case Western Reserve University School of Medicine, Cleveland, OH 44106 USA; 2 Laboratory of Cellular and Molecular Biology, National Institute of Diabetes and Digestive and Kidney Diseases, National Institutes of Health, Bethesda, MD 20892 USA; 3 Department of Pharmacology, Case Western Reserve University School of Medicine, Cleveland, OH 44106 USA; 4 Center for Mitochondrial Diseases, Case Western Reserve University School of Medicine, Cleveland, OH 44106 USA; 5 Frances Payne Bolton School of Nursing, Case Western Reserve University, Cleveland, OH 44106 USA

**Keywords:** Biochemistry, Mitochondrial proteins, CNS cancer, Glycobiology, Post-translational modifications, Post-translational modifications, Organelles, Mitochondria

## Abstract

*O*-GlcNAcylation is a prevalent form of glycosylation that regulates proteins within the cytosol, nucleus, and mitochondria. The *O*-GlcNAc modification can affect protein cellular localization, function, and signaling interactions. The specific impact of *O*-GlcNAcylation on mitochondrial morphology and function has been elusive. In this manuscript, the role of *O*-GlcNAcylation on mitochondrial fission, oxidative phosphorylation (Oxphos), and the activity of electron transport chain (ETC) complexes were evaluated. In a cellular environment with hyper *O*-GlcNAcylation due to the deletion of *O*-GlcNAcase (OGA), mitochondria showed a dramatic reduction in size and a corresponding increase in number and total mitochondrial mass. Because of the increased mitochondrial content, OGA knockout cells exhibited comparable coupled mitochondrial Oxphos and ATP levels when compared to WT cells. However, we observed reduced protein levels for complex I and II when comparing normalized mitochondrial content and reduced linked activity for complexes I and III when examining individual ETC complex activities. In assessing mitochondrial fission, we observed increased amounts of *O*-GlcNAcylated dynamin-related protein 1 (Drp1) in cells genetically null for OGA and in glioblastoma cells. Individual regions of Drp1 were evaluated for *O*-GlcNAc modifications, and we found that this post-translational modification (PTM) was not limited to the previously characterized residues in the variable domain (VD). Additional modification sites are predicted in the GTPase domain, which may influence enzyme activity. Collectively, these results highlight the impact of *O*-GlcNAcylation on mitochondrial dynamics and ETC function and mimic the changes that may occur during glucose toxicity from hyperglycemia.

## Introduction

*O*-GlcNAcylation is a ubiquitous post-translational modification (PTM) that is tightly regulated by two enzymes, *O*-GlcNAc transferase (OGT) and *O*-GlcNAcase (OGA), that add and remove the sugar modification respectively^1^. Serine and threonine residues are modified through covalent attachment of the *O*-GlcNAc moiety. Often, these sites can be phosphorylated, which creates PTM crosstalk between O-GlcNAcylation and phosphorylation that subsequently impacts protein function and cell regulatory pathways^2–4^. This regulation through *O*-GlcNAc cycling may impact metabolic status in response to nutrients and substrate availability. As such, dysregulation of *O*-GlcNAc cycling can give rise to the etiology of aging associated with chronic diseases, including cardiovascular diseases, cancer, diabetes, metabolic disorders, and neurodegenerative diseases^1,5,6^.

Mitochondria are essential metabolic organelles that control the production of cellular energy in the form of adenosine triphosphate (ATP). These membrane bound compartments respond to cellular cues to regulate their activity, and physical changes are observed in parallel with functional changes. In fact, mitochondria are dynamic organelles that undergo cycles of fusion and fission in response to cellular signaling, including changes in cellular energy demands, nutrient stress, and during apoptotic progression^7^. Moreover, these opposing processes sustain organelle homeostasis through mitochondrial dynamics that mix organelle contents and isolate damage. An imbalance in mitochondrial fission or fusion is known to contribute to several diseases mentioned above, including cancer, neurodegenerative diseases, diabetes, and cardiovascular diseases^1,6,8–10^.

The proteins that regulate mitochondrial dynamics are within the same dynamin superfamily^11^. Outer membrane fusion requires mitofusin 1 (MFN1) and mitofusin 2 (MFN2), which unite opposing mitochondrial membranes. Optic atrophy 1 (OPA1) connects the inner mitochondrial membranes and maintains cristae morphology. In opposition to these proteins, dynamin-related protein-1 (Drp1) executes outer membrane division. Drp1 is an 80 kDa cytosolic protein that is actively recruited to the surface of mitochondria to promote membrane remodeling. This process is tightly regulated through lipid and protein interactions as well as a diverse collection of post-translational modifications, including phosphorylation, nitrosylation, ubiquitylation, sumoylation, and *O*-GlcNAcylation^12–27^. Phosphorylation sites are the most well-characterized PTMs that regulate mitochondrial dynamics^28^. In fact, several kinases and phosphatases have been shown to alter Drp1 function through both activating and inactivating phosphorylations^19–21,29^. Nitrosylation was observed in neurodegenerative disorders and augments mitochondrial fission^23,30^. Drp1 *O*-GlcNAcylation was characterized in cardiac myocytes of diabetic animals and when a small molecule inhibitor of OGA was added, a subtle increase in mitochondrial fission was observed^26^.

Mitochondrial morphology reflects changes in cell physiology through various signaling pathways that respond to cellular cues, including nutrient status, cell cycle control and cell health. Excessive mitochondrial fission or fusion leads to an imbalance in mitochondrial dynamics. Additionally, changes in mitochondrial morphology have been implicated in bioenergetic changes^31,32^**.** While organelle elongation is proposed to enhance electron transport chain (ETC) activity, increased fission limits oxidative phosphorylation (Oxphos)^33^. This may be due to changes in cristae morphology or other differences in membrane architecture that alter assembly of individual ETC complexes and larger respiratory supercomplexes^34,35^. It was initially found that small matrix volume, or fragmented mitochondria, equated to increased matrix electron concentration in primary liver cells^36^. Later, it was observed that interconnected mitochondria coincided with higher metabolic activity within energy demanding cells, while fragmented mitochondria have diminished respiratory activity^37^. Still, there is significant variability in mitochondrial morphology and subsequent impact on its functional capacity^31,32,38^. The precise role of PTMs in regulating mitochondrial morphology and function still remains unknown because of the complex, interconnected nature of signaling pathways. Moreover, the indirect consequences of small molecules that alter PTM signaling and the diversity of assays used to assess changes in mitochondrial function have complicated interpretations.

This manuscript explores the impact of the *O*-GlcNAc modification on mitochondrial ultrastructure and function. Our results demonstrate the impact of a genetic deletion induced global hyper-*O*-GlcNAcylation cellular environment on Drp1 *O*-GlcNAc modifications, changes in mitochondrial morphology, mitochondrial Oxphos and activity of ETC complexes not seen in previous studies using pharmacologic inhibitors. We found that selective removal of OGA to enhance cellular *O*-GlcNAcylation augments mitochondrial fission, leading to diminished activity of select ETC complexes. To sustain energy demands through mitochondrial activity, these cells exhibit a compensatory increase in mitochondrial mass to recover diminished respiration. These results highlight the impact of hyperglycemic disease states on mitochondrial physiology in response to altered *O*-GlcNAc cycling.

## Results

### Mitochondrial fission is enhanced in OGA KO MEFs

**Figure 1:**
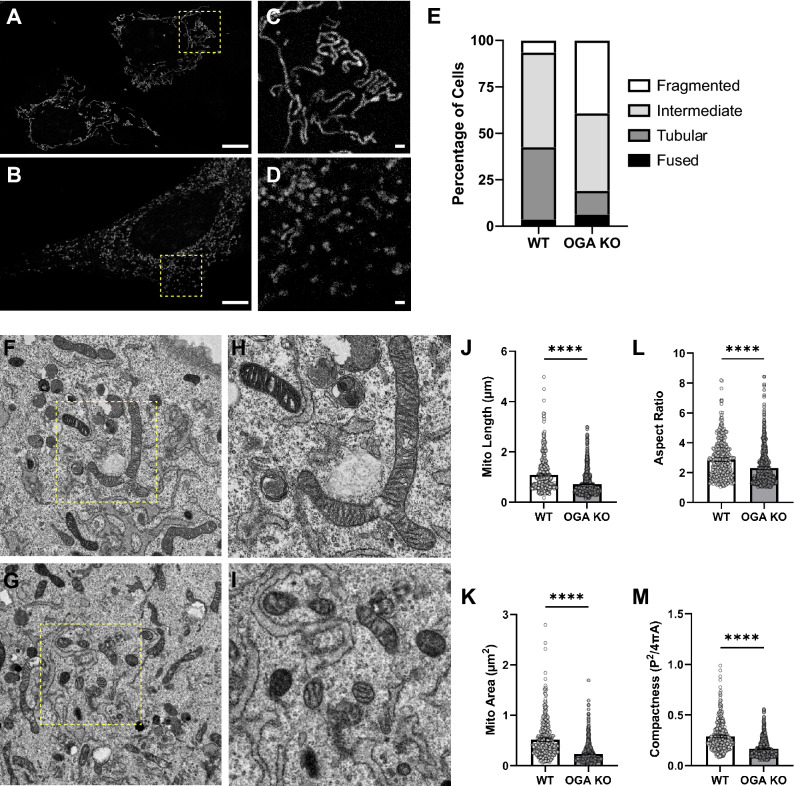
Mitochondrial Fission is Enhanced in OGA KO MEFs. (**A–D**) Confocal microscopy images with Mitotracker dye are presented for WT (**A**) and OGA KO (**B**) MEFs; Scale bars, 10 µm. Select areas (highlighted with a yellow dashed box) are enlarged for WT (**C**) and OGA KO (**D**) MEFs; Scale bars, 1 µm. (**E**) The quantified percentage of cells characterized by mitochondrial morphologies (fused, tubular, intermediate, or fragmented) in basal WT and OGA KO MEFs are shown (WT cells = 108 counted, OGA KO cells = 110 counted). (**F–I**) Representative transmission electron microscopy (TEM) images of fixed sections for WT (**F**) and OGA KO (**G**) MEFs are presented; Scale bar, 2 µm. Enlarged areas of WT (**H**) and OGA KO (**I**) MEFs highlight local mitochondrial morphologies. Quantification of mitochondrial length (J), area (K), aspect ratio (L), and compactness (M) are shown (An unpaired Student’s t test shows ****p < 0.0001; WT mitochondria = 391 counts, OGA KO mitochondria = 616 counts).

To explore the role of *O*-GlcNAc signaling on mitochondrial function and structure, we utilized SV-40 large T antigen transformed OGA knockout (KO) mouse embryonic fibroblasts (MEFs) that exhibit a global hyper-*O*-GlcNAcylated cellular environment^39,40^. Previous studies have examined the effect of *O*-GlcNAc signaling using chemical inhibitors in different disease models. Initially, we sought to determine how genetic deletion of OGA, which alters cycling of the modification, impacts the mitochondrial dynamics using different imaging methods. First, we performed confocal imaging on basal WT and OGA KO MEFs stained with MitoTracker Orange and scored mitochondrial morphology within each cell as either fused, tubular, fragmented, or an intermediate state. We found that mitochondrial morphology was predominantly intermediate and tubular in the basal WT MEFs, making up 51% and 39% of measured mitochondria in these cells respectively (Fig. 1A,C,E). Fragmented and fused morphologies were rare in WT MEFs (4% and 6% respectively). In comparison, the morphology of mitochondria within the OGA KO MEFs exhibited a shift towards intermediate and fragmented states, which made up 42% and 39% of the cells respectively. Fused and tubular morphologies were rare in the OGA KO MEFs (6% and 13% respectively; Fig. 1B,D,E), consistent with an increase in mitochondrial fission when OGA was not present.

To complement the clear shift in mitochondrial morphology observed by confocal imaging, we analyzed the size and shape of mitochondria using transmission electron microscopy (TEM) on fixed sections of cultured cells (Fig. 1F–I). A quantitative assessment showed that the mitochondria were clearly more elongated in the WT MEFs when compared to the OGA KO MEFs. The measured lengths shifted from an average of 1.01 µm in the WT cells to 0.72 µm in the KOs (Fig. 1J). This change is also reflected in the Aspect Ratio (length vs width) of the mitochondria, with the average ratio of 2.9 in WT compared to 2.3 in cells lacking OGA (Fig. 1L). The biggest difference was observed in the measured area for mitochondria in each cell. The average area was roughly halved in the KO cells compared to control (0.24 µm^2^ in the KO vs. 0.52 µm^2^ in the WT; Fig. 1K). In these images, the mitochondria did not appear rounded in the hyper *O*-GlcNAcylated cells, and the overall appearance of the organelles seemed relatively normal; they were just smaller. A final assessment was to measure the compactness of the mitochondrial structure, which highlights a relationship between the perimeter of the compartment and the area (Perimeter^2^/4π*Area). Again, the KO cells were found to have more compact mitochondrial shapes when compared to the WT cells (average values were 0.17 in the KO vs. 0.29 in the WT; Fig. 1M). Collectively, these data demonstrate the effect of *O*-GlcNAc cycling on mitochondrial ultrastructure and a smaller organelle morphology was evident in the hyper- *O*-GlcNAcylated cellular environment.

Interestingly, the number of mitochondria in each cell was dramatically elevated in the OGA KO MEFs. From the electron microscopy analysis, the total number of mitochondria was counted with within WT and OGA KO MEFs for dozens of cells. On average, 6.4 mitochondria were found within an area of 100 µm^2^ of WT cells (403 within a total measured area of 6,300 µm^2^). Comparatively, we found 19.3 mitochondria per 100 µm^2^ in OGA KO cells (739 counted within a total measured area of 3825 µm^2^). OGA KO cells were observed to be uniformly larger than the matched WT cells, which leads to a dramatic increase in the number of mitochondria per cell consistent with the differences observed by confocal imaging (Fig. 1A vs. B). This increase in mitochondrial number, and potentially mitochondrial mass, were hypothesized to contribute to changes in organelle function.

### Oxidative respiration and phosphorylation are largely preserved in a hyper *O-*GlcNAcylated cellular environment

**Figure 2:**
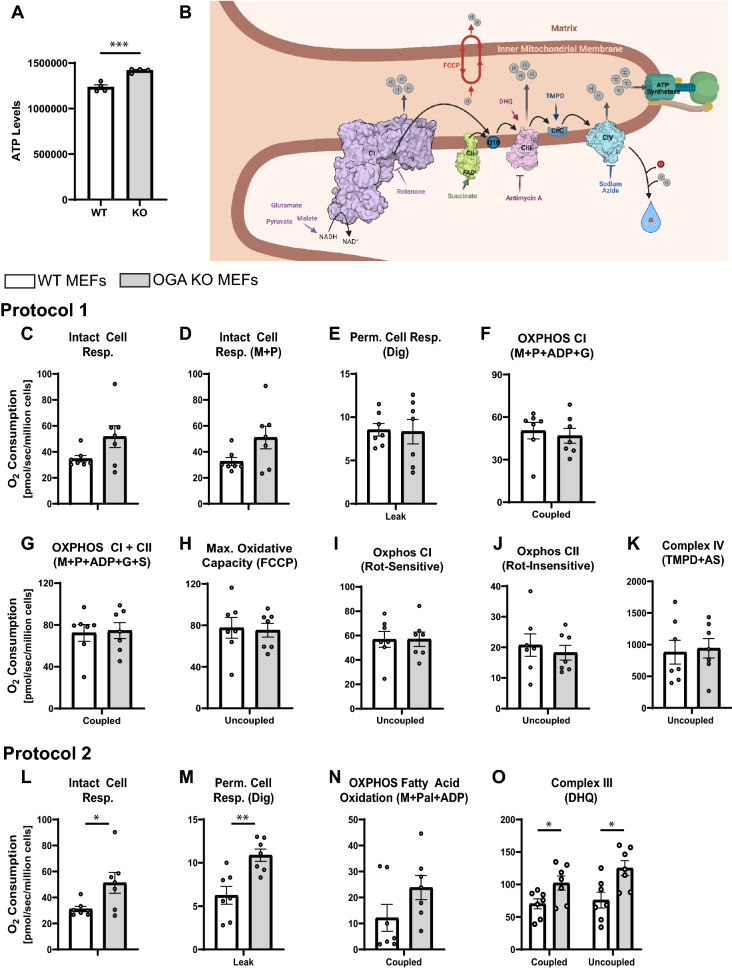
Comparison of Oxidative Phosphorylation in permeabilized WT and OGA KO cells. (**A**) Relative ATP levels were measured in WT and OGA KO MEFs. (**B**) A schematic representation of the individual ETC complexes is shown highlighting the substrates and inhibitors used in Protocols 1 and 2. (**C–O**) The measured oxygen consumption rates (pmol O_2_/sec/million cells) observed for WT (white bars) and OGA KO (grey bars) MEFs treated with the indicated substrates or inhibitor are presented. For Protocol 1 (**C**–**K**), intact cellular respiration (ICR) rate (**C**), ICR with malate (M) and pyruvate (P) (**D**), Digitonin (Dig) Permeabilized Cell Respiration, coupled leak rate (**E**), Oxphos of CI (M + P + adenosine diphosphate [ADP] + G), coupled (**F**), Oxphos of CI + CII (M + P + ADP + G + S), coupledF (**G**), Maximal Oxphos Capacity (trifluoromethoxy carbonylcyanide phenylhydrazone [FCCP] added, panel (**H**), Oxphos of CI (Rot-sensitive), uncoupled (**I**) Oxphos of CII (Rot-insensitive), uncoupled (**J**), and Oxphos of CIV (TMPD + AS), uncoupled (**K**) were determined. For Protocol 2 (**L**–**O**), ICR rate (**L**), Digitonin (Dig) Permeabilized Cell Respiration, coupled leak rate (**M**), Oxphos of fatty acid (M + palmitoylcarnitine [Pal] + ADP), coupled (**N**), Oxphos of CIII (duroquinol [DHQ]), coupled and uncoupled (**O**) were measured (An unpaired Student’s t test shows *p < 0.05; **p < 0.01). Representative traces for WT and OGA KO MEFs are presented in Supplementary Figure S1.

The observed differences in mitochondrial morphologies suggested that the impaired *O*-GlcNAc cycling and increased *O*-GlcNAcylation in the OGA KO cellular environment shifts the mitochondrial dynamics and morphology towards fragmentation. We asked whether these changes in mitochondrial morphology alter the overall bioenergetic capacity of the mitochondria in these cells. We assessed ATP levels in a comparable number of WT and OGA KO MEFs and observed a 15% increase in ATP within the OGA KO MEFs (Fig. 2A). This showed that cellular ATP was maintained despite differences in mitochondrial morphology.

Therefore, we sought to ask whether OxPhos was altered in this cellular environment. To assess the functional differences in mitochondria between WT and OGA KO MEFs, we measured Oxphos within intact, permeabilized cells using established techniques^41^. This method includes two protocols run in parallel^41–43^, using a number of selective substrates and inhibitors to measure the rates of oxygen consumption attributed distinct ETC complexes (Fig. 2; tracings Sup. Fig. S1). In protocol 1 (Fig. 2C–K), the activities of complexes I, II, and IV are sequentially assessed and measured. First, intact cellular respiration (ICR) is determined to obtain a baseline rate prior to the addition of any substrates. Interestingly, we found that the ICR rate was slightly higher in the OGA KO MEFs, which is consistent with the cellular ATP measurements and suggested that both WT and OGA KO cells had at least equivalent baseline Oxphos (Fig. 2C). Then, substrates for Complex I (CI), malate (M) and pyruvate (P), were added, but elicit no impact on the cellular respiration as these substrates remain extracellular (Fig. 2D). To permeabilize the cell membrane, digitonin (Dig) was added, resulting in diluted intracellular content and allowing M + P to enter the cell for oxidation, which reveals the leak rate (Fig. 2E). Adenosine diphosphate (ADP) was then added, to achieve state 3, followed by glutamate (G), another CI substrate, and succinate (S), a complex II substrate. These gave an assessment of the coupled CI Oxphos and coupled CI + CII Oxphos (Fig. 2F,G). To assess the maximal oxidative capacity, the uncoupler, trifluoromethoxy carbonylcyanide phenylhydrazone (FCCP), was added and showed an increased oxygen consumption rate more than 30% over the ADP-stimulated rates of coupled CI and coupled CI + CII (Fig. 2H). Next, the addition of rotenone (Rot), an inhibitor of CI, assesses the uncoupled CI and CII rates individually. The absolute diminishment of the FCCP rate by Rot is attributed as the uncoupled CI activity (Rot-sensitive rate; Fig. 2I) and the remaining respiration represents the uncoupled CII activity (Rot-insensitive rate; Fig. 2J). Lastly, the addition of antimycin A (AA), a complex III inhibitor, provides an assessment of non-mitochondrial respiration. To assess complex CIV Oxphos, we added tetramethyl-p-phenylenediamine (TMPD) and ascorbate (AS), which donates electrons to cytochrome *c* (cyt *c*), then a CIV inhibitor, azide, was added (Fig. 2K). Our assessment of complexes I, II, and IV in the OGA KO MEFs revealed comparable integrated complex activities, suggesting that mitochondrial Oxphos was sustained in the hyper-*O*-GlcNAcylated cellular environment.

In protocol 2, we measured the Oxphos for fatty acid oxidation (FAO) and complex III (CIII). The ICR rate was first assessed (Fig. 2L) followed by the addition of M and palmitoylcarnitine (Pal), a long-chain fatty acid derivative. Similar to protocol one, the leak rate was obtained with the addition of Dig (Fig. 2M). After that, ADP was added to assess the state 3 rate of Pal oxidation (Fig. 2N). Then Rot was added to diminish CI activity, which is followed by adding duroquinol (DHQ), a CIII substrate to assess the coupled CIII rate (Fig. 2O). Finally, the uncoupled CIII rate was evaluated via the addition of FCCP. We found that the ICR rate, the FAO rate, and coupled CIII activity trended higher in the OGA KO MEFs, while the leak rate and uncoupled CIII oxidation were significantly higher in these hyper *O*-GlcNAcylated cells.

Collectively, the results from both protocols show that the increase in mitochondrial content in the OGA KO MEFs sustains the Oxphos capacity for these cells despite significant structural remodeling of this organelle network. Previous studies had shown changes in the functional activity of individual ETC complexes due to *O*-GlcNAc modifications^33,44–46^. Since we did not see any substantial changes at the cellular level, we decided to evaluate the activity of individual, isolated complexes using established protocols^42,47–49^. The significant increase in uncoupled CIII respiration in protocol 2 may reflect differences better observed in ETC complex activity. Moreover, the increase in mitochondrial content that we saw through imaging suggests that more mitochondrial mass is required to sustain ETC activity in the cell.

### Individual ETC complexes are affected in a hyper-*O*-GlcNAcylated environment

**Figure 3:**
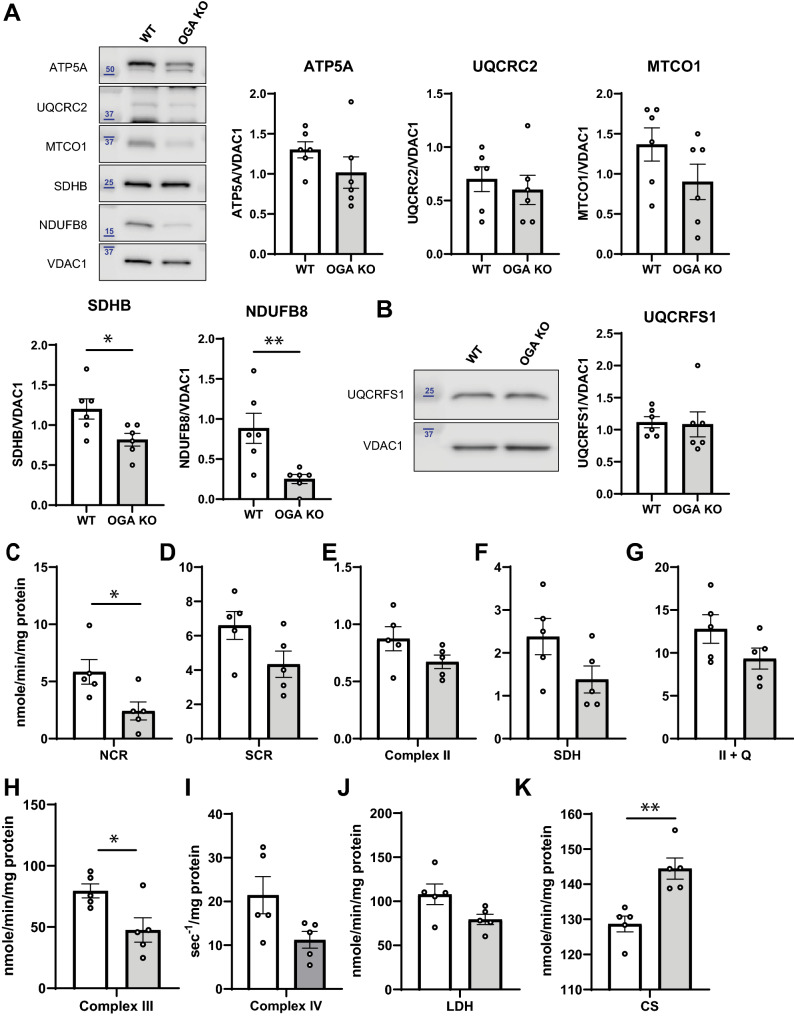
Decreased ETC complex protein content and activities in OGA KO MEFs. (**A**) ETC proteins content were detected by western blot and quantified relative to VDAC1; Complex I = NDUFB8 (NADH:Ubiquinone Oxidoreductase Subunit B8, Complex II = SDHB (Succinate Dehydrogenase Complex Iron Sulfur Subunit B), Complex III = UQCRC2 (Ubiquinol-cytochrome *c* reductase core protein 2), Complex IV = MTCO1 (Mitochondrially Encoded Cytochrome *C* Oxidase I), ATPase Synthase = ATP5A (ATP Synthase F1 Subunit Alpha). (**B**) The protein content of UQCRFS1 (Ubiquinol-Cytochrome *C* Reductase, Rieske Iron-Sulfur Polypeptide 1) was also assessed. Blue numbers indicate molecular weight markers. (**C**–**K**) The individual complex rates (nmol/min/mg protein) were measured for CI + CIII (**C**) rotenone-sensitive NADH cytochrome *c* reductase, NCR), CII + CIII (**D**) antimycin-sensitive succinate cytochrome *c* reductase, SCR), the Thenoyltrifluoroacetone (TTFA)-sensitive complex II (**E**, Complex II), succinate dehydrogenase (**F**, SDH), complex II with exogenous coenzyme Q analogue added (**G**, CII + Q), decylubiquinol-cytochrome *c* oxidoreductase (**H**, Complex III), and lactate dehydrogenase (**I**, LDH). The first order rate constant (k-^1^/mg protein) for Complex IV was determined (**J**). The activity of citrate synthase (CS) was also measured (**K**). An unpaired Student’s t test shows *p < 0.05; **p < 0.01. Full-length Western Blots (uncropped) are provided in Supplementary Figure S2.

While the mitochondrial Oxphos was preserved when assessing the intact cellular physiology, we hypothesized that isolated ETC complex activity may exhibit some deficiency because of the observed increase in mitochondria number per cell. Using a cocktail containing primary antibodies against individual proteins in each ETC complex, we were able to measure their relative abundance. From these Western blots (Fig. 3A), the amount of each protein was normalized to VDAC1, an outer mitochondrial membrane protein control. The ATP synthase was unaffected as ATP5A content were not significantly different. However, both complexes I and II protein abundance were significantly diminished in the OGA KO MEFs. The NDUFB8 protein amount in the OGA KO was less than half that observed in the WT, and the SDHB content was decreased by ~ 30% in cells with hyper *O*-GlcNAcylation. A similar trend was seen for CIV (MTCO1), but the measured difference was not significant. Interestingly, CIII (UQCRC2) content was similar on average. Since all of the other complexes exhibited a downward trend in protein content, we used a separate antibody against the Rieske iron-sulfur polypeptide of CIII (UQCRFS1; Fig. 3B). Again, we did not see any diminution in the amount of CIII based on protein content.

Next, the activities of individual ETC complexes were assessed^42,47–49^, with rates normalized to cellular protein amounts (Fig. 3C–K). Each ETC complex showed decreases in activity, and large differences were found in the linked activity of complexes I + III (NCR; Fig. 3C) and complexes II + III (SCR; Fig. 3D). Specifically, the NCR dropped from 5.2 to 2.2 nmol cyt *c* reduced/min/mg protein and the SCR dropped from 6.2 to 4.0 nmol cyt *c* reduced/min/mg protein when comparing WT and OGA KO cells, respectively. These changes are consistent with the decreased content for proteins in CI and CII (Fig. 3A). Similarly we observed, decreases in Thenoyltrifluoroacetone (TTFA)-sensitive CII (from 0.87 to 0.67 nmol DCPIP reduced/min/mg protein; Fig. 3E) and CII with added exogenous coenzyme Q analogue (from 12.8 to 9.3 nmol DCPIP reduced/min/mg protein; Fig. 3G) when comparing the WT and OGA KO MEFs. Moreover, the first-order CIV activity was reduced almost twofold (from 21.4 to 11.2 k^-1^/mg protein; Fig. 3I). The activity of decylubiquinol-cytochrome *c* oxidoreductase (CIII) was also significantly reduced (from 79.5 to 47.6 nmol cyt *c* reduced/min/mg protein; Fig. 3H), highlighting a significant decrease in CIII activity even though the protein amounts for this complex were not altered. Based on these findings, the activities of ETC complexes I, II, III, and IV were diminished in the OGA KO cells.

Consistent with the TEM results, the citrate synthase (CS) amounts were elevated in the OGA KO cells, even when normalized to the total cellular protein content (Fig. 3K). Again, this likely reflects an increase in mitochondrial number and mass in a hyper-*O*-GlcNAcylated cellular environment (Fig. 1), which is able to sustain the bioenergetic demands for the OGA KO cell despite clear defects within their individual mitochondrial ETC complexes (Fig. 3C-J).

### Drp1 *O*-GlcNAcylation is elevated in OGA KO MEFs, glioblastoma (GBM) cell lines, and sustained on overexpressed Drp1 mutants

**Figure 4:**
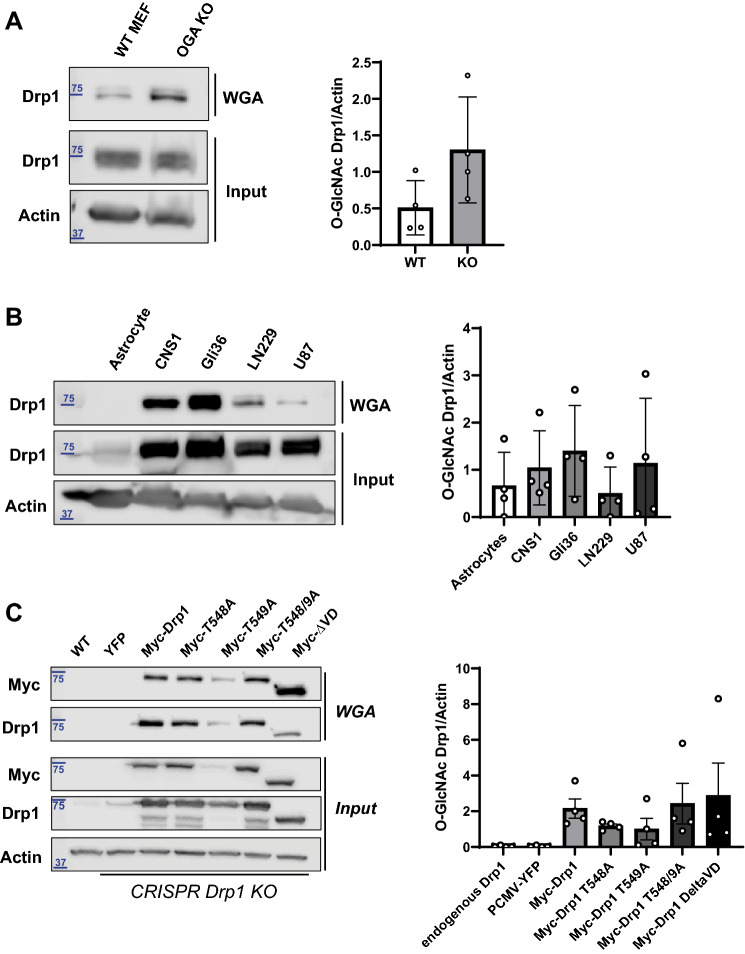
Drp1 *O*-GlcNAcylation is elevated in OGA KO MEFs, glioblastoma (GBM) cell lines, and sustained on overexpressed Drp1 mutants. (**A**) Western blot analysis of Drp1 within WGA precipitates from WT and OGA KO MEFs is shown and quantified. N = 6, Student’s t test, error bars represent SEM. (**B**) Western blot analysis of Drp1 from WGA precipitation of human astrocytes and glioblastoma cell lines (CNS1, Gli36, LN229, and U87) is presented with quantification. N = 4, Student’s t test, error bars represent SEM. Input is 10% protein concentration of precipitation. (**C**) Western blots of Myc-tagged Drp1 (WT, T548A, T549A, T548/9A, and ΔVD) constructs overexpressed and WGA precipitated from Drp1 CRISPR HCT 116 cells is shown and quantified. N = 4, Student’s t test, error bars represent SEM. Input is 10% protein concentration of precipitation. Full-length Western Blots (uncropped) are provided in Supplementary Figure S3.

Based on the ultrastructural and functional changes that we observed in OGA KO MEFs compared to control, we sought to examine the molecular changes contributing to the enhanced mitochondrial fission. In previous studies, *O*-GlcNAcylation of Drp1 was detected in neonatal rat cardiomyocytes using immunoprecipitation with an *O*-GlcNAc antibody (RL2) and subsequently probing with an antibody against Drp1^26^. Furthermore, the use of a small molecule inhibitor of OGA enhanced this modification. We asked whether genetic deletion of OGA would exhibit a similar pattern. To avoid complications from the peptide backbone interactions with *O*-GlcNAc antibodies^50^, we used wheat germ agglutinin (WGA) coated agarose beads to precipitate the *O*-GlcNAc sugar moiety independent of peptide sequence^51^. Using WT and OGA KO MEFs, we were able to consistently precipitate *O*-GlcNAc-modified Drp1 using WGA beads (Fig. 4A), and we found that impaired cycling of *O*-GlcNAc through the loss of OGA resulted in a detectable increase in *O*-GlcNAcylated Drp1.

To highlight the relevance of this modification in a disease state, we used the same method to isolate *O*-GlcNAcylated Drp1 in four GBM cell lines (rat, CNS1, and human, Gli36, LN229, and U87) and compared the abundance of modified protein with amounts in healthy astrocytes (Fig. 4B). The amount of modified protein was relatively low in astrocytes, while the abundance of *O*-GlcNAc modified Drp1 was elevated in the GBM models. Therefore, Drp1 *O*-GlcNAcylation occurs across different species and in aggressive cancer cells. The detectable elevation in *O*-GlcNAcylation of Drp1 in the GBM cell lines compared to the healthy astrocyte control suggests that there is an increase in *O*-GlcNAcylation, reflecting physiologic changes in this cancer. Increased mitochondrial fission has been implicated as a contributing factor to GBM tumor recurrence^52^, and *O*-GlcNAc cycling may contribute to this behavior.

**Figure 5:**
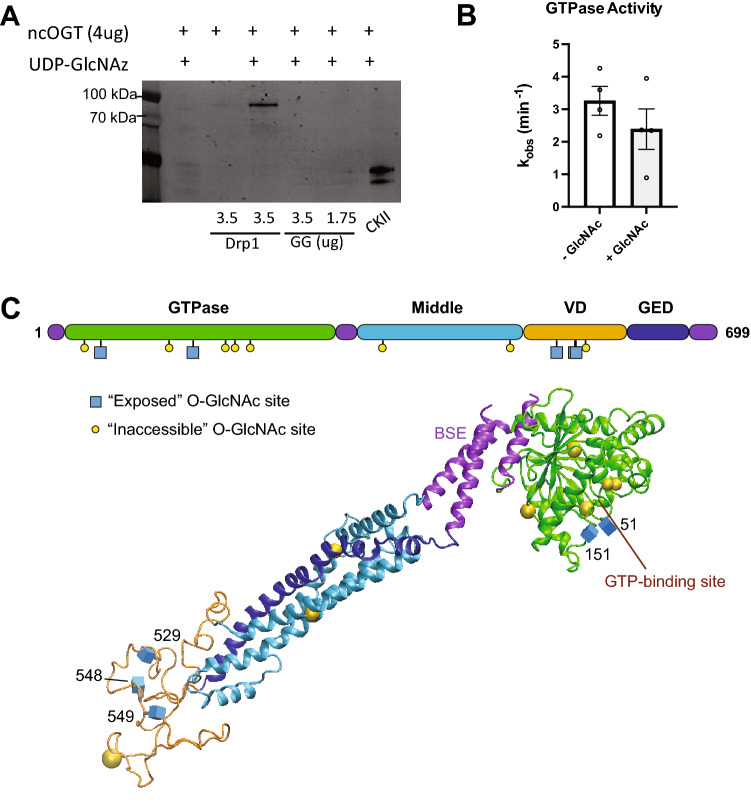
In vitro validation and Prediction of Drp1 *O*-GlcNAcylation Sites. (**A**) In vitro* O*-GlcNAcylation of recombinant Drp1 and Drp1 GTPase-GED (GG, GTPase domain, green, & BSE, magenta purple) with recombinant OGT was observed. (**B**) GTPase activity rates were measured using a continuous GTPase assay with full-length Drp1 that had been incubated with OGT with or without UDP-GlcNAc. (**C**) Drp1 protein domain schematic and a 3D homology model highlights predicted *O*-GlcNAc modifications (blue cubes denote solvent accessible predicted residues and yellow spheres denote solvent inaccessible predicted residues). The complete list of predicted sites is available in Supplementary Table 1.

The variable domain (VD) of Drp1 is a common site of regulation as different PTMs, including phosphorylation, ubiquitination and SUMOylation, have been identified within this region. Similarly, two *O*-GlcNAc modified sites were identified at threonine residues, T585 and T586, using mass spectrophotometric analyses of Drp1 from cardiomyocytes^26^. The VD is also the major site for alternative splicing in Drp1, and these sequence differences can lead to alternate numbering. Specifically, residues T585 and T586 within a longer Drp1 isoform containing alternative exons (NCBI Accession Code: NP_036192.2) correspond to T548 and T549 in the variant used in these studies (Fig. 5; NCBI Accession Code: NP_005681.2). *O*-GlcNAcylation of these neighboring threonines within the VD of Drp1 were shown to enhance translocation of Drp1 from the cytosol to the mitochondria, which is consistent with the regulatory role of the VD in Drp1 self-assembly and membrane interactions^16,26^. An increase in GTP binding was also observed with *O*-GlcNAcylated Drp1, suggesting a change in the GTPase domain, which is on the opposing side of the Drp1 molecule based on structures from X-ray crystallography^53^. Therefore, we asked whether additional sites in the protein could be modified by OGT.

To assess whether defined sites of *O*-GlcNAcylation in Drp1 were exclusive, we made alanine mutations at T548 and T549 (equivalent to T585 and T586 in the longer isoform) in a mammalian Myc-tagged Drp1 expression construct, which was introduced to HCT116 cells lacking endogenous Drp1 due to a CRISPR knockout^54^. WGA was used to precipitate glycosylated proteins and an anti-Myc antibody probed for over-expressed Drp1 that had been modified. We found that mutations at T548 and T549 separately and in combination did not prevent *O*-GlcNAc modification of Drp1 as the Myc-tagged constructs were still precipitated by WGA (Fig. 4C lanes 4–6). This suggested that Drp1 has additional *O*-GlcNAc sites beyond the two described previously in cardiomyocytes.

In an attempt to further diminish *O*-GlcNAcylation of Drp1, we used a construct lacking the entire VD (∆517–639), since this regulatory region is subjected to multiple post-translational modifications. When using WGA, we were still able to precipitate the truncated Drp1 (∆VD, Fig. 4C lane 7). This finding reveals that Drp1 *O*-GlcNAcylation is not limited to the VD and must be present in the GTPase and/or stalk regions of Drp1. Collectively, our results indicate a more extensive distribution of *O*-GlcNAc modification on Drp1 in vivo than previously reported.

### In vitro validation and prediction of Drp1 *O*-GlcNAcylation sites

To further confirm that Drp1 is *O*-GlcNAc modified, we performed an in vitro assay in which recombinant OGT was incubated with recombinant Drp1 in the presence of the bioorthoginal sugar UDP-GlcNAz, followed by a staudinger ligation as previously described^55^. In vitro modification provides insight to a protein’s ability to be modified in vivo since confirmed, in vivo OGT substrates have also been modified with this assay, including casein kinase II^56,57^. The ability of OGT to modify Drp1 was compared with casein kinase II as an established positive control (Fig. 5A lane 6). We found that Drp1 was modified to a similar extent as the control, which confirms that Drp1 can be *O*-GlcNAcylated directly as a substrate of OGT (Fig. 5A lane 3).

The GTPase and VD sites are on opposing ends of the molecule, and labeling of these regions would explain the different impacts of *O*-GlcNAc modification on Drp1 self-assembly and GTP binding. To see whether the GTPase domain alone could be modified directly by OGT, we isolated a previously characterized GTPase-GED (GG) fusion construct. This soluble fragment of Drp1 was incubated with recombinant OGT to assess *O*-GlcNAc modifications using the same method described above. We did not detect the modification using this smaller construct (Fig. 5A lanes 4 and 5). Taken together, these results suggest that the sites in the middle domain may be accessible and modified in vivo and that modification of the GTPase domain in vitro may require the complete protein structure for effective protein recognition by OGT. It is also possible that an unknown cofactor may be required in vitro for modification of the GTPase domain.

Given these findings, we used several *O*-GlcNAcylation prediction tools (OGTSite, YingOYang, DictyOGlyc1.1, & Glycam) to predict additional Drp1 *O*-GlcNAcylation sites (Supplementary Table 1). Of the 40 serines and 42 threonines within the shorter Drp1 isoform, thirteen sites were predicted to be modified by OGT using three prediction tools, OGTSite, YingOYang, and DictyOGlyc 1.1. (a similar assessment was done with the longer isoform, and 4 additional sites were identified in the alternative sequence in the VD, see Supplementary Table 2). Four predicted sites had overlapping predictions, including T185 and S194 within the GTPase domain, T479 in the middle domain, and the previously identified T549 within the VD. To further refine our search, the solvent accessible surface area (SASA) of each site was predicted using GlyCam website (www.glycam.org), which is calculated by NACCESS (http://www.bioinf.manchester.ac.uk/naccess/). With this additional restriction, five sites were predicted to be accessible to OGT for *O*-GlcNAc modification, including two sites in the GTPase domain (T55 and T151) and three sites in the VD (S529 and the previously identified T548 and T549). To highlight the distribution of the solvent accessible predicted sites, *O*-GlcNAc moieties were modeled onto a homology model that was generated using the complete Drp1 sequence (Fig. 5C). The structure is largely based on the X-ray structure of Drp1^53^, which lacks the entire VD and is missing a few small loops. The VD is predicted to be intrinsically disordered, which can be seen with the lack of secondary structure in this region (orange ribbon, Fig. 5C). This also means that the fourth predicted site in the VD (S559) is likely to be accessible in a different conformation. This was a limitation of making predictions with a static structure, and subtle motions could expose additional sites, which would be consistent with a broader distribution of *O*-GlcNAc modification in Drp1.

To further probe the effect of *O*-GlcNAc modification on Drp1 function, a continuous GTPase assay was used to measure the relative enzyme rates of proteins that were modified by UDP-GlcNAc. A mild decrease in Drp1 activity was observed in the treated sample when compared to the untreated protein (Fig. 5B). This decrease may underestimate the full impact of *O*-GlcNAc modification on Drp1 activity because the in vitro modification does not yield complete modification of the protein. Still, these results demonstrate the functional impact of *O*-GlcNAc modification on Drp1 activity. While a decrease in activity might be expected to limit fission, GTP hydrolysis also promotes Drp1 disassembly^58^. Therefore, the observed decrease in activity for the modified protein may promote assembly of the mitochondrial fission machinery to enhance its role in membrane remodeling. Moreover, this mild change in activity is consistent with other PTM changes that do not drastically alter enzyme activity. Rather, these change fine-tune the assembly properties of Drp1 to regulate mitochondrial fission.

## Discussion

**Figure 6:**
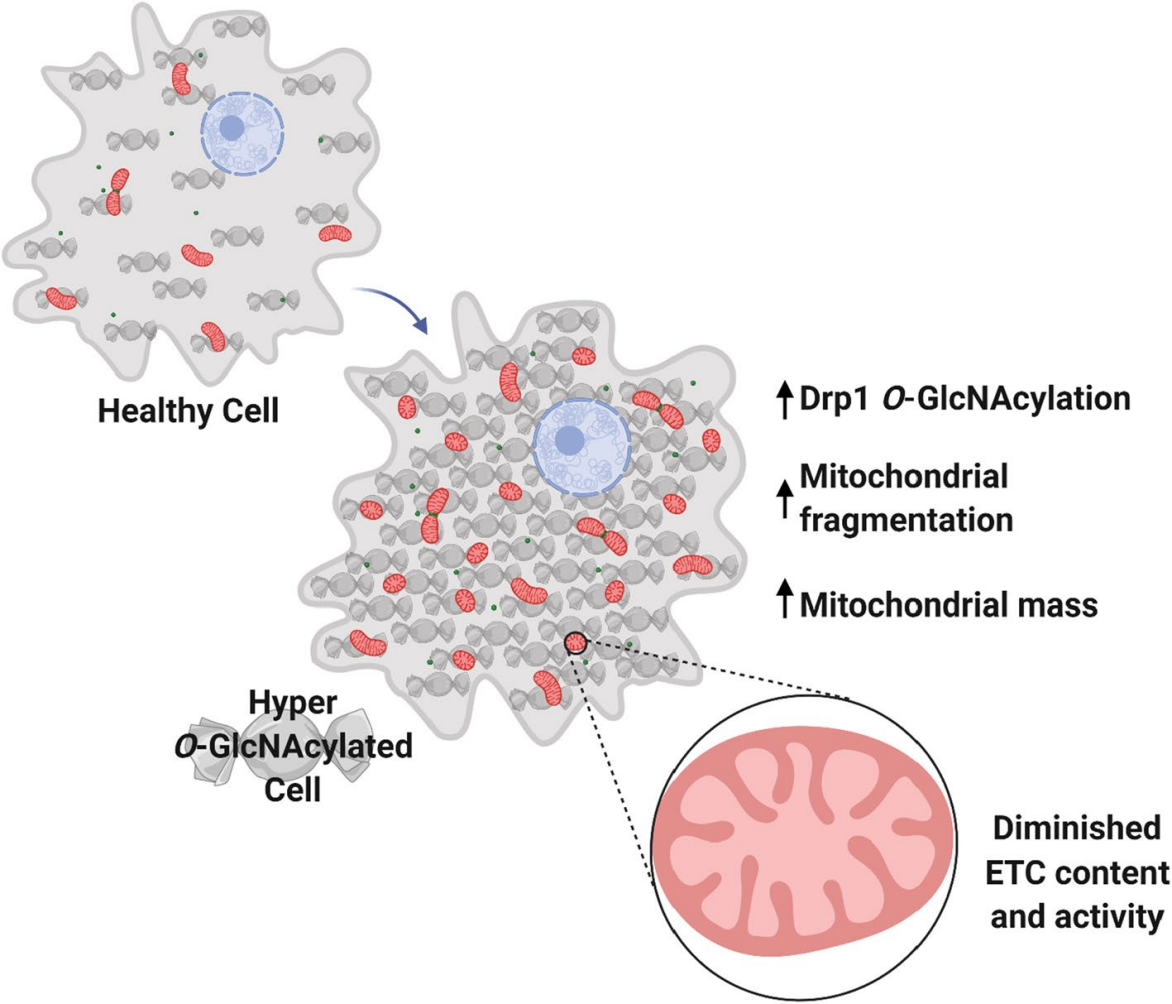
Model of the Effect of Hyper-*O*-GlcNAcylation on Mitochondrial Structure and Function. Elevated *O*-GlcNAcylation (represented by candies) within the cellular environment leads to mitochondrial fragmentation (executed by Drp1, green dots) and diminished ETC content and activity. A compensatory increase in mitochondrial mass compensates for these local defects, leading to relatively normal respiratory capacity when compared to a healthy cell with proper *O*-GlcNAc cycling. Model created with BioRender.com.

Impaired *O*-GlcNAc cycling skewed mitochondrial dynamics and results in altered morphology, which is consistent with several disease states, including cancer, diabetes, and cardiovascular disease (Fig. 6). The relationship between these morphological changes and mitochondrial function are not always clear. Surprisingly, our studies revealed that coupled oxidative phosphorylation is largely unaffected within the OGA KO MEFs as intact cellular respiration and ATP levels were slightly elevated. However, there were significant decreases in protein amounts within complex I and II upon closer examination of the individual ETC complexes. Previously, proteins in complex I, III and IV were shown to be *O*-GlcNAcylated, leading to impaired activity in a hyper-glycemic environment^44^. We identified a decrease in proteins in ETC complexes I, II, and IV, but we did not see any change in the core proteins of complex III (Fig. 3; UQCRC2 and UQCRFS1 were unchanged). Therefore, *O*-GlcNAcylation does not alter protein stability of this complex, but the activity was significantly diminished (Fig. 3H), which suggests that CIII may be regulated by this modification by a distinct mechanism when compared to other ETC complexes. To this point, we demonstrated that complex II protein amounts decrease in a hyper-O-GlcNAcylated environment, similar to complex I. It is possible that transcription and/or protein stability is decreased for these complexes, but more studies are needed to elucidate this mechanism. Nevertheless, complexes with diminished protein content (I, II and IV) in OGA KO MEFs all exhibited decreased average enzyme activity.

In the OGA KO MEFs, we suggest that the increase in mitochondrial number and content, based on imaging and citrate synthase activity, is a compensatory response to overcome the respiratory limitations of ETC complexes in a hyper-*O*-GlcNAcylated cellular environment (Fig. 6). Within a disease state where altered *O-*GlcNAc cycling is sustained for an extended period of time, resulting in chronic disease such as hyperglycemia, it has been shown that mitochondrial function is impaired^59^. Specifically, excessive *O*-GlcNAcylation has been suggested to impair mitochondrial function^44^. Our research aligns with these findings, but the compensatory increase in mitochondrial content seems to stabilize respiratory capacity despite having smaller organelle compartments. Specifically, the mitochondrial number and density in the KO cells tripled when compared to WT MEFs (6.4 per 100 µm^2^ in WT vs. 19.3 per 100 µm^2^ in OGA KO), so despite a 50% decrease in the size (area) of the mitochondria, the overall content increased. ATP content has been reported to be diminished in hyperglycemic conditions^44^, but our results found no decrease in ATP levels, suggesting that increased mitochondrial number can rescue bioenergetic changes in a hyper-*O*-GlcNAcylated state. Still, the efficiency of individual ETC complexes is diminished, and future studies will better define the alterations to *O*-GlcNAc modification of individual ETC proteins and the underlying reason for altered protein amounts when OGT activity is unopposed. A less efficient respiratory chain may lead to increased oxidative stress, and accumulation of damage to mitochondrial DNA in this disease state^60^. Chronic exposure to this type of environment likely leads to other defects in mitochondrial function that limit cellular responses to stress. In fact, excessive mitochondrial fission is associated with apoptosis in several degenerative disorders and during hyperglycemia^61^, and inhibition of organelle fission restored morphology and prevented cell death. As such, inhibition of mitochondrial fission may partially rescue defects seen in a hyperglycemic state.

The observed increase in mitochondrial fission in OGA KO MEFs may reflect increase biogenesis or mitophagy to remove damage. In both cases, the decrease in size can limit the bioenergetic capacity, so an increase in mitochondrial number is required to sustain cellular energy, and the intact cell respiration was found to be unaltered when comparing the WT and OGA KO MEFs (Fig. 2). The key proteins that govern mitochondrial dynamics must be altered to produce the changes in ultrastructure. In these studies, we show that Drp1 *O*-GlcNAcylation is elevated in cells lacking the enzyme that removes this modification. We also found that this Drp1 modification was increased in GBM cell lines, demonstrating altered Drp1 glycosylation in this type of cancer. Precipitation with WGA coated agarose beads and in vitro OGT assays with UDP-GlcNAz were able to detect modified proteins, overcoming limitations associated with *O*-GlcNAc antibodies^50,51,62–65^. Further, we demonstrated that Drp1 *O*-GlcNAcylation likely extends beyond its variable domain and modifications have the potential to be more distributed at multiple sites within its structure. Unfortunately, the *O*-GlcNAc moiety was too labile to withstand mass spectrometry analysis, the best method to definitively map the modified sites of Drp1 *O*-GlcNAcylation^51^. Our attempts to detect the *O*-GlcNAc moiety on endogenous and recombinant in vitro modified protein via CTD/ETC techniques were unsuccessful. This led to our use of computational tools, trained on established *O*-GlcNAc modified protein, to aid in predicting specific sites of modification on Drp1, and we identified 16 potential sites in the long form of Drp1 including alternative exons (Supplementary Table 2). Identification of *O*-GlcNAc modified proteins has advanced substantially from the first use of purified glycosyltransferases to probe for exposed saccharide moieties on the surface of intact cells in 1983^51^.

Drp1 is tightly regulated by PTMs, and often the total protein content is not different when comparing healthy and diseased states. Drp1 can be acetylated, phosphorylated, sumoylated, ubiquitinated, S-nitrosylated, and *O*-GlcNAcylated. The impact that these modifications have on Drp1 activity is largely understudied, but differences in Drp1 localization within the cell have been noted. Phosphorylation of Drp1 at S616 activates the recruitment to the outer mitochondrial membrane, leading to increased fission. Conversely, S637 phosphorylation is inhibitory and restricts localization largely to the cytosol, which limits mitochondrial fission. *O*-GlcNAcylation is thought to exhibit crosstalk with phosphorylation because the two amino acids that can be *O*-GlcNAcylated, serine and threonine, can also be phosphorylated. But there is currently no overlap with Drp1 *O*-GlcNAcylation sites predicted in this study and any characterized phosphorylation sites. The crosstalk between these PTMs and the concomitant impact on Drp1’s localization, assembly status, and enzymatic function still remains to be elucidated. However, our findings show that within cells with elevated *O*-GlcNAc levels, mitochondrial fission is increased, which is accompanied with decreased mitochondrial length, compactness, aspect ratio, and area. Since the percentage of fused mitochondria in the WT MEFs is similar to that in the OGA KO MEFs (Fig. 1E), mitochondrial fusion appears to be undisturbed. Therefore, the mechanism by which Drp1 *O*-GlcNAcylation serves as an activating PTM may be through opposition to S637 phosphorylation and promoting S616 phosphorylation. Indeed, the modification appears to diminish the GTPase activity of Drp1, suggesting that *O*-GlcNAc regulation of mitochondrial fission may be through augmented assembly or interactions with partner proteins, which may be directly or indirectly related to phosphorylation status.

The VD of Drp1 is an intrinsically disordered region, and recent results have shown that it serves a regulatory role in self-assembly of the mitochondrial fission machinery. Post-transcriptionally, the VD can be alternatively spliced, producing various isoforms of Drp1. Additionally, this region is the primary site of PTMs that tightly regulate Drp1 functional assembly. This impacts intra- and inter-molecular interactions that govern cellular localization and membrane remodeling through the formation of large helical complexes. In this way, the VD is a natural site to regulate Drp1 function. While several residues in this region are predicted to be *O*-GlcNAc modified, the removal of this domain did not prevent WGA precipitation. While we could not reconstitute the *O*-GlcNAc modification with the truncated Drp1 G-domain, the GTPase activity of the full length protein was impaired when the modification was present. This is consistent with the position of solvent exposed G domain residues that are predicted to be modified directly adjacent to the nucleotide binding site in Drp1 (Fig. 5C). Thr55 and Thr151 are proximal to Switch I and II, respectively, which coordinate phosphate binding and hydrolysis. The addition of one or more GlcNAc moieties in this region would likely impair nucleotide stabilization and hydrolysis, consistent with our findings and previous results that suggest nucleotide binding is impaired^16^.

Collectively, our results suggest that blocking O-GlcNAc cycling has a dramatic effect on mitochondrial number, mass, and activity. Although we have completely blocked O-GlcNAc removal, the effects of hyper-O-GlcNAcylation are likely to reflect a physiological function of this modification in mitochondrial maintenance.

## Methods

### Antibodies

Antibodies included Actin (Sigma Aldrich, A5441), RL2 (Thermo Fisher Scientific, MA1072), CTD110.6 (Cell Signaling, CST-9875), HGAC85 (Novus, NB300614), c-Myc (Santa Cruz, sc40), OGA (Abcam, ab124807), OGT (Abcam, ab177941), Drp1 (Santa Cruz, sc101270), and GAPDH (Cell Signaling, CST-2118) OxPhos Cocktail (Abcam, ab110413), VDAC1 (Abcam, ab154856), UQCRFS1 (Abcam, ab1474).

### Cell lines

WT and OGA KO Murine embryonic fibroblasts (MEFs) as previously described^40^ were cultured in DMEM with 10% FBS. Glioblastoma cell lines CNS1 derived from rat (cultured in RPMI with 10% FBS), Gli36 derived from human (cultured in DMEM with 10% FBS), LN229 derived from human (cultured in DMEM with 5% FBS), and U87 derived from human (cultured in DMEM with 10% FBS) supplemented with 5% L-Glutamine and 5% penicillin–streptomycin. HCT116 and HCT116 CRISPR Drp1 cells cultured in McCoy’s medium supplemented with 10% FBS.

### Immunoblotting

Cells were collected and lysed in 1 × RIPA lysis buffer (Cell Signaling, CST-9806) supplemented with [0.1% Sodium dodecyl sulfate (Sigma Aldrich, 436143), 0.1% Lauryl-beta-D-maltoside (Sigma-Aldrich, D4641), 0.5% Triton (ACROS, 21568–2500), 1 × PhosSTOP Phosphatase Inhibitor (ROCHE, 04906837001), 1 × cOmplete Mini Protease Inhibitor Cocktail (ROCHE, 11836153001), and 100 µM PugNAc (Toronto Research Chemicals, A157250)]. Lysed cells were incubated on ice for 30 min and centrifuged at 16,000×*g* at 4 ºC for 20 min. Protein concentration was determined using the Pierce™ BCA Protein Assay Kit (Thermo Fisher Scientific, 23228). Equal amounts of protein (30 µg) were mixed with reducing Laemmli loading buffer (BIORAD, 1610747), boiled and electrophoresed on hand poured acrylamide gels, then transferred to PVDF membranes (BIORAD, 1620177). Blocking was performed for 20–45 min with 5% dry milk (Nestle, 274200) in 1 × TBST and blotting performed with primary antibodies at 1:500–1000 dilutions overnight (16–18 h) at 4 ºC while rocking. Primary antibodies were detected with species specific HRP conjugated secondary antibodies. Specifically, Amersham ECL Prime Western Blotting Detection Reagent (Cytiva) for 2–3 min prior to image acquisition. Densitometry quantification of bands were performed as^66^ using ImageJ.

### Wheat germ agglutinin affinity isolation

Detection of Drp1 *O*-GlcNAcylation was performed as outlined in^51^. Briefly, Wheat Germ Agglutinin coated Agarose beads (Vector Laboratories,al-1023) were incubated overnight with protein lysate to lectin precipitate *O*-GlcNAc modified protein. Unbound protein was washed three times in PBS-N+ (0.2% NP-40, 1× protease and phosphatase inhibitor cocktails, plus PugNAc), bead and bound protein was boiled in laemmli buffer and run on an acrylamide gel as outlined in Immunoblotting protocol. Detailed analysis of protein content were done via ImageJ as mentioned in^66,67^.

### Transfection of WT and Mutant Drp1 constructs

Transfection of specified constructs (8ug of YFP, WT Drp1, Drp1 T548A, Drp1 T549A, Drp1 T548/9A, Drp1 delta VD within the pCMV backbone) were performed with Lipofectamine 2000 (Therm Fisher Scientific, 1166819) per the manufacturer’s protocol.

### Prediction of Drp1 *O*-GlcNAc sites

The complete Drp1 sequences for isoforms 1 and 3 (NCBI Accession Codes NP_036192.2 and NP_005681.2, respectively) were used to assess all possible sites of *O*-GlcNAcylation using OGTSite, YingOYang, and DictyOGlyc 1.1 prediction sites. A list was compiled and Glycam was used to evaluate solvent accessibility (SASA) using a homology model based on the Drp1 crystal structure (PDB ID: 4BEJ). Based on the combination of solvent accessibility and sequence identification from the prediction sites, proposed *O*-GlcNAc modifications were modeled using the 3D implementation of the Symbol Nomenclature for Graphical Representation of Glycans (3D-SNFG) plugin^68^ in VMD^69^.

### Purification of recombinant Drp1

Drp1 (isoform 3) was expressed and purified as described previously^70^. Briefly, Drp1 constructs were overexpressed in BL21-(DE3) Star E. *coli*, purified by calmodulin affinity purification, the CBP-tag was removed with HVR3C protease, and size-exclusion chromatography was used to achieve full sample purity. The Drp1 GG construct was kindly provided by the Ramachandran lab. Expression was performed in BL21-(DE3) Star E. *coli* and the protein was purified using NTA(Ni^2+^) agarose affinity purification, cleaned up with ion exchange chromatography, and purified to homogeneity with size-exclusion chromatography as described in^71^.

### In vitro *O*-GlcNAcylation assay

In vitro modification of recombinant Drp1 (80 KDa) via recombinant OGT was performed and thoroughly described in^55^. Briefly, OGT glycosyl transferase reactions were performed using the indicated amount of recombinant ncOGT, 2 mM UDP-GlcNAz and the indicated substrate in buffer containing 50 mM tris–HCl, pH 7.5, 1 mM DTT, 12.5 mM MgCl_2_ at 37 ºC for ~ 100 min. Reactions were then centrifuged for 30 min in 10 K filters at 14,000 rpm, washed with PBS twice, and recovered. A Staudinger ligation was then performed on the recovered samples by incubating with 1 µl biotin phosphine (30 mM stock) for 60 min at 40 ºC. Reactions were then boiled in loading dye for 10 min, stored in -80 ºC overnight. Samples were run on a 4–12% nuPAGE gel (Thermo Fisher), transferred to a nitrocellulose membrane, blocked with odyssey blocking buffer (Licor) probed with 1:10000 IRDye 800CW streptavidin (Licor) and visualized on a Licor Odyssey system.

### Continuous GTPase assay

We used an assay in which GTP is continuously regenerated in order to measure the enzymatic activity of WT Drp1 with and without the *O*-GlcNAc modification. This assay was performed as described previously^72^, but with a few modifications. The assay was completed at 37 °C and all components were pre-warmed to 37 °C prior to beginning the assay. Each reaction contained 200 nM Drp1 protein, 25 mM HEPES(KOH) pH 7.5, 150 mM KCl, 10 mM BME, 5 mM MgCl_2_, 1 mM phospho(enol) pyruvate (Sigma P7002), 600 µM NADH, approximately 20 units/mL pyruvate kinase/lactate dehydrogenase mixture (Sigma P0294), and 500 µM GTP. The solution containing GTP was added last to start the reaction. The absorbance at 340 nm was measured with a VICTOR *X*3 Multimode Plate Reader (PerkinElmer). The absorbance was measured at 10 s intervals for 50 min in total. Data was analyzed in Microsoft Excel and the NADH depletion over time was used to calculate the rate of GTP hydrolysis.

### Immunofluorescent staining, confocal imaging, and quantification

Cells were plated on glass-bottom dishes and were stained with 250 nm of Mitotracker orange CMTMRos (Invitrogen, M7510) for 30 min then fixed in 4% paraformaldehyde (Alfa Aesar, 43368) for 15 min and stained with DAPI in PBS 1:10,000 (Invitrogen, D1306) for 5 min. 2–3 washes were performed between each step to reduce background. Images acquired using a Leica SP8 Gated STED confocal Microscope in the Light Microscopy Imaging Facility at Case Western Reserve University. Blinded individuals independently categorized each cell as either fused, tubular, intermediate, or fragmented for randomized confocal images. The number of cells in each category were divided by the total number of cells imaged to calculate the percentage of each category in WT or OGA KO MEFs. These data were compiled into Prism 9 (GraphPad Software, LLC) for statistical analysis and graphical representation.

### Transmission electron microscopy

Cells were seeded on the Corning® Costar® Transwell® cell culture inserts. When cells were 80–90% confluent, they were then washed with PBS and fixed in 2% glutaraldehyde/0.1 M sodium cacodylate buffer (pH 7.4) at room temperature for 1 h. Cells were washed twice in PBS and postfixed in 2% OsO4/PBS at room temperature for 1 h, dehydrated in ethanol and embedded in Epon (Serva, Heidelberg, Germany). Thin sections of 50 nm were contrasted with uranylacetate and lead citrate, and examined using a FEI Tecnai 12 transmission electron microscope equipped with a Gatan single tilt holder and a Gatan US4000 4kx4k CCD camera. All images were assessed and morphological measurements determined using ImageJ^73^. These data were imported into Prism 9 software for statistical analysis and graphical representation.

### ATP assay

As briefly described previously^52^, the assessment of cell viability in the readout of luminescence which is directly proportional to intracellular ATP levels was performed using the CellTiter-Glo® Luminescent Cell Viability Assay (Promega, G7571). Experiment was performed as clearly outlined per the manufacturer’s protocol (easily searchable on Google). Succinctly, CellTiter-Glo® Reagent is added to carefully counted cells and shaken orbitally, incubated for 10 min, and read within a luminometer.

### Oroboros measurement of respiration within permeabilized cells

Oxygen consumption was measured with an O2K system (OROBOROS) using Protocols 1 and 2 described in^41^. Briefly, 2 ml of cell suspension (600,000 cells/ml) in Mir05 buffer was added to each chamber. Digitonin was added in increments (2ug/ml) to permeabilize the cell membranes. The final concentration of substrates and inhibitors was composed of malate (2 mM), pyruvate (2.5 mM), adenosine diphosphate (ADP, 2.5 mM), glutamate (10 mM), succinate (10 mM), palmitoylcarnitine (10 µM), duroquinol (0.5 mM), tetramethyl-p-phenylenediamine (TMPD, 0.5 µM), ascorbate (2 mM), carbonylcyanide-p-trifluoromethoxy- phenylhydrazone (FCCP, 0.5-µM increment), rotenone (75 nM), antimycin A (125 nM), and sodium azide (200 mM). For Protocol 1, the non-mitochondrial (AA-insensitive) rate is subtracted. For Protocol 2, the Rot-insensitive rate is subtracted. For DHQ, the AA is subtracted.

### ETC activity assays

As described previously^49^, MEFs were pelleted and resuspended at 4 °C in 750 µl of MSM/2 mM EDTA buffer (220 mM mannitol, 70 mM sucrose, 5 mM MOPS, pH 7.4, and 0.1 M EDTA) with 7.5 µl of proteinase inhibitor (Sigma- Aldrich, Cat # P8340) and kept on ice. Protein concentration was determined by the Lowry assay using bovine serum album as a standard^74^ and the sample prepared using 5% cholate and MSM/2 mM EDTA at 1 mg/ml of protein for assays except for cytochrome *c* oxidase^75^. A second sample for cytochrome *c* oxidase was prepared in MSM/2 mM EDTA at 1 mg/ml of protein without cholate^76,77^.

ETC enzyme activity measurement, the methods used in the Hoppel lab were published previously^42,47,48^. Enzymatic activities of complexes I, II, III, and IV in murine embryonic fibroblasts (MEFs) were determined using spectrophotometry. Rotenone-sensitive NADH cytochrome *c* reductase was used to measure the linked activity of complex I and III and antimycin A-sensitive succinate cytochrome *c* reductase was used to measure the linked activity of complexes II and III. Complex II activity was measured as the thenoyltrifluoroacetone (TTFA)-sensitive succinate-2,6 Dichlorophenol-indophenol (DCPIP) reductase and total complex II determined in the same way but with Coenzyme Q1 added^42^. Succinate dehydrogenase (SDH) was used to measure the first 2 subunits (A and B) of complex II and served as a membrane-bonded mitochondrial marker enzyme. In addition to SDH, we measured citrate synthase activity as the other mitochondrial marker, as well as the activity of lactate dehydrogenase (LDH) as cytoplasmic marker. Antimycin A-sensitive decylubiquinol cytochrome *c* reductase (complex III) was utilized to determine the reduction of cytochrome *c* coupled to the oxidation of decylubiquinol to decylubiquinone. Complex III was inhibited by antimycin A and the assay was measured as an antimycin A-sensitive component. Cytochrome *c* oxidase (complex IV) was measured as the first order rate constant.

## Supplementary Information

Supplementary Information.
