# Supplementary material for: Blocked O-GlcNAc cycling alters mitochondrial morphology, function, and mass
Source: Sci Rep. 2021 Nov 11;11:22106. doi: 10.1038/s41598-021-01512-y (PMC8586252; doi:10.1038/s41598-021-01512-y)

Supplementary Information

for

Blocked *O*-GlcNAc cycling alters mitochondrial morphology, function, and mass

Elizabeth O. Akinbiyi[1], Lara K. Abramowitz[2], Brianna L. Bauer[3], Maria S.K. Stoll[4], Charles L. Hoppel[3,4], Chao-Pin Hsiao[4,5], John A. Hanover[2], and Jason A. Mears[3,4,*]

[1] Department of Pathology, Case Western Reserve University School of Medicine, Cleveland, OH 44106

[2] Laboratory of Cellular and Molecular Biology, National Institute of Diabetes and Digestive and Kidney Diseases, National Institute of Health, Bethesda, MD, 20892, USA

[3] Department of Pharmacology, Case Western Reserve University School of Medicine, Cleveland, OH 44106

[4] Center for Mitochondrial Diseases, Case Western Reserve University School of Medicine, Cleveland, OH 44106

[5] Frances Payne Bolton School of Nursing, Case Western Reserve University, Cleveland, OH 44106

[*] To whom correspondence should be addressed: jason.mears@case.edu

Supplementary Table 1. Comprehensive Prediction of O-GlcNAc Sites in Drp1 Isoform 3

	Residue	Sequence	SASA1	Prediction Resource		
				OGTSite[2]	YingOYang[3]	DictyOGlyc 1.1[4]
	36	VGTQSSGKSSV	0.2 -		+	
■	**55**	LLPRGTGIVTR	**119.6 +**	+		
	126	NNKGVSPEPIH	12.4 -			+
■	**151**	DLPGMTKVPVG	**50.4 +**			+
	185	IILAVTAANTD	11.4 -	+	+	
	194	TDMATSEALKI	11.2 -	+	+	
	210	PDGRRTLAVIT	1.4 -	+		
	347	TEYCNTIEGTA	11.1 -	+		
■	479	KRLPVTNEMVH	0.0 -	+	+	
■	**529**	**LPSAVSRDKVA**	**22.6**		+	
■	***548**	**GVQEPTTGNWR**	**75.7 +**	+		
■	***549**	**VQEPTTGNWRG**	**34.4**	+	+	
	559	GMLKTSKAEEL	11.6 -	+		

[1]Solvent Accessible surface Area for Amino Acid from Glycam site (www.glycam.org)

Site predictions identified from [2]OGTSite (http://csb.cse.yzu.edu.tw/OGTSite/), [3]YingOYang (http://www.cbs.dtu.dk/services/YinOYang/), and [4]DictyOGlyc 1.1 (http://www.cbs.dtu.dk/services/DictyOGlyc/)

*Residues 548 and 549 were idenitied in a previous study (Gawlowski *et al.*)

Supplementary Table 2. Comprehensive Prediction of O-GlcNAc Sites in Drp1 Isoform 1

	Residue	Sequence	SASA[1]	Prediction Resource		
				OGTSite[2]	YingOYang[3]	DictyOGlyc 1.1[4]
	36	TQSSGKS	0.2 -		+	
■	**55**	PRGTGIV	**119.6 +**	+		
	126	KGVSPEP	12.4 -			+
■	**151**	PGMTKVP	**50.4 +**			+
	185	LAVTAAN	11.4 -	+	+	
	194	MATSEAL	11.2 -	+	+	
	210	GRRTLAV	1.4 -	+		
	347	YCNTIEG	11.1 -	+		
■	**479**	LPVTNEM	**0.0 -**	+	+	
■	**529**	SAVSRDK	**22.6**		+	
■	**534**	DKSSKVP	**50.7 +**		+	
	544	APASQEP	13.2 -		+	
■	**548**	QEPSPAA	**40.6 +**		+	
■	***585**	QEPTTGN	**75.7 +**	+		
■	***586**	EPTTGNW	**34.4**	+	+	
	596	LKTSKAE	11.6 -	+		

[1]Solvent Accessible surface Area for Amino Acid from Glycam site (www.glycam.org)

Site predictions identified from [2]OGTSite (http://csb.cse.yzu.edu.tw/OGTSite/), [3]YingOYang (http://www.cbs.dtu.dk/services/YinOYang/), and [4]DictyOGlyc 1.1 (http://www.cbs.dtu.dk/services/DictyOGlyc/)

*Residues 585 and 586 were idenitied in a previous study (Gawlowski *et al.*)

Supplementary Figure S1. Representative Traces of O2K Analyses.

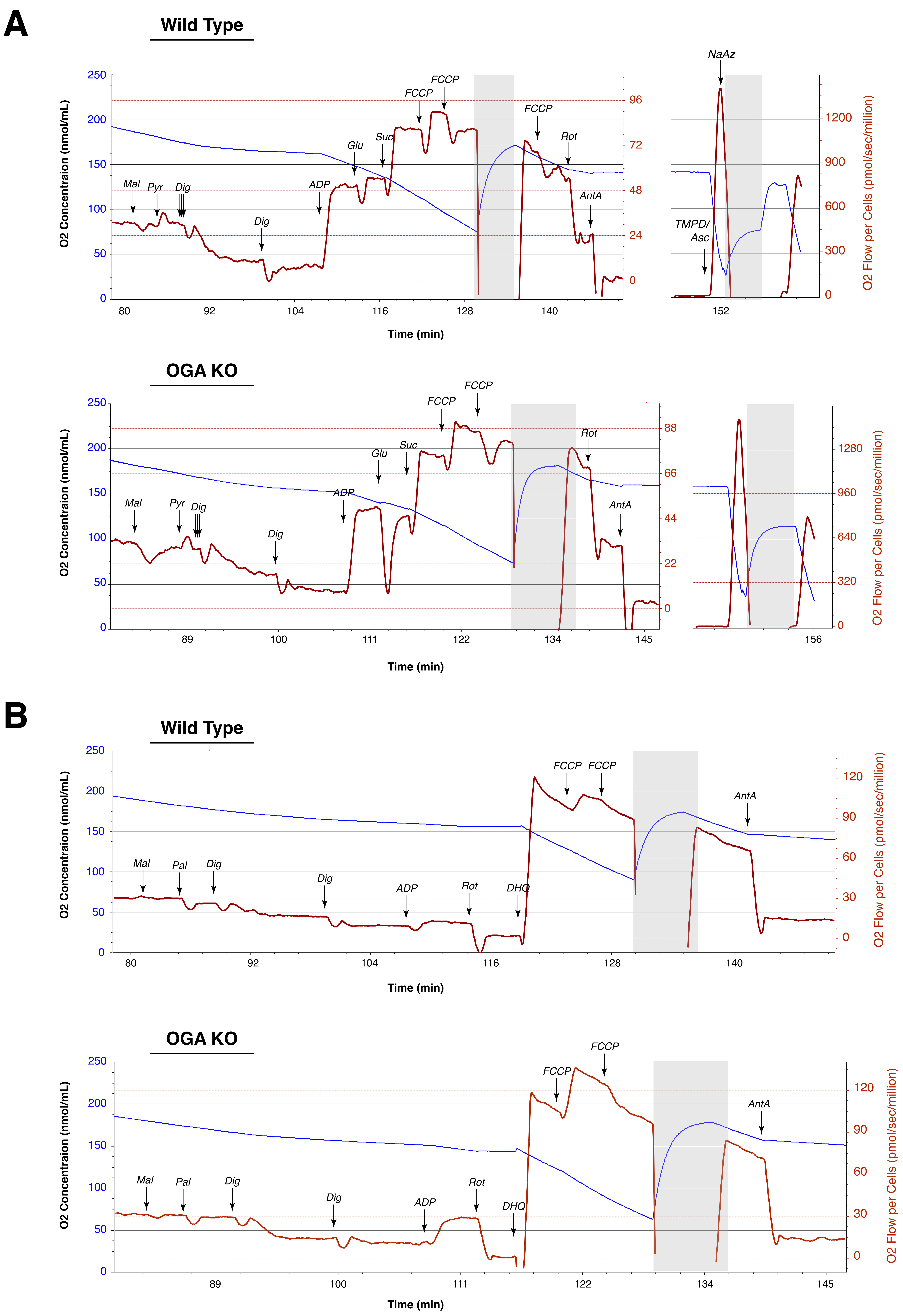

Supplementary Figure S2.

Figure 3B Blot probing UQCRFS1

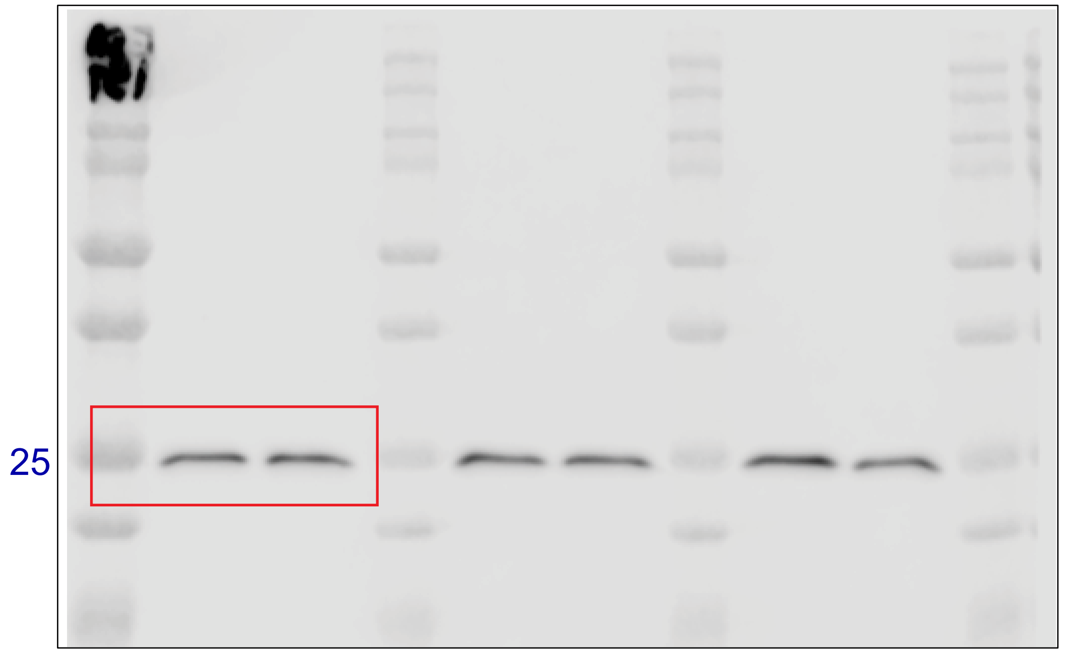

Figure 3B Blot probing VDAC1

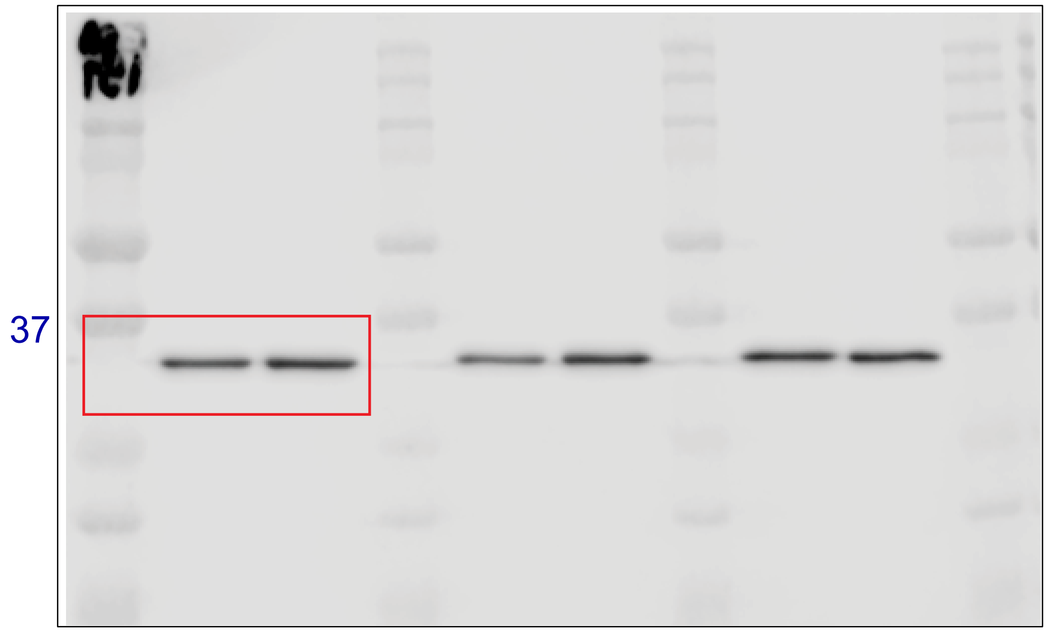

Figure 3A Blot probing ETC Cocktail

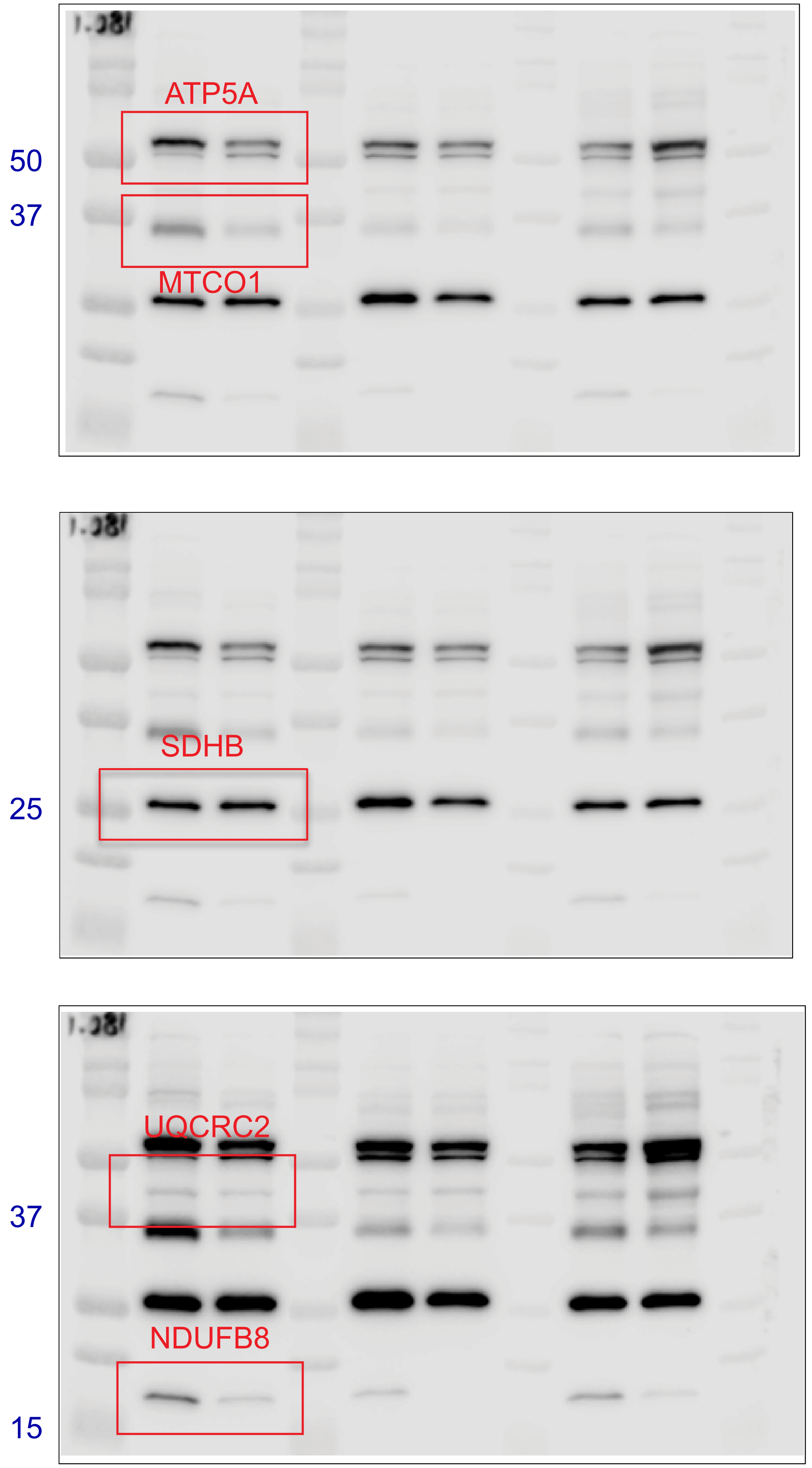

Figure 3A Blot probing VDAC1

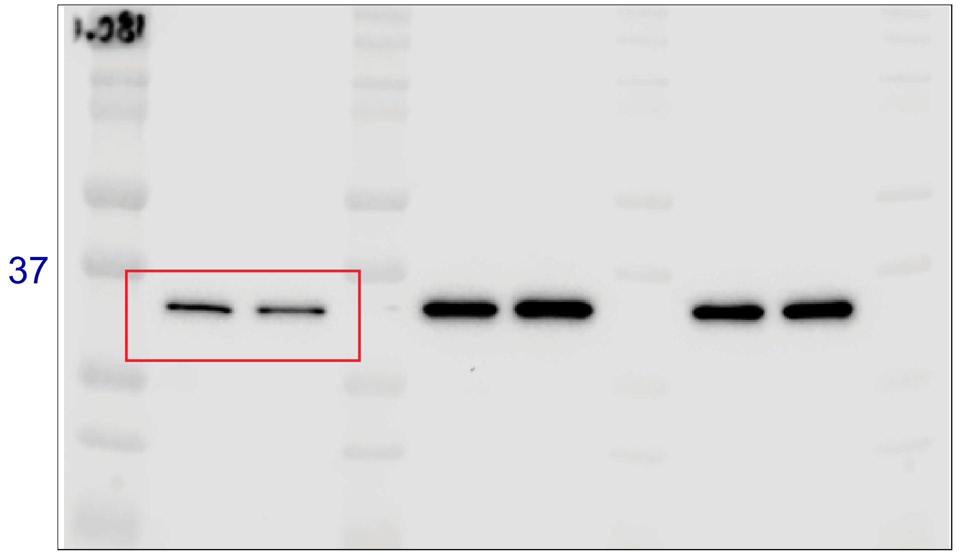

Supplementary Figure S3.

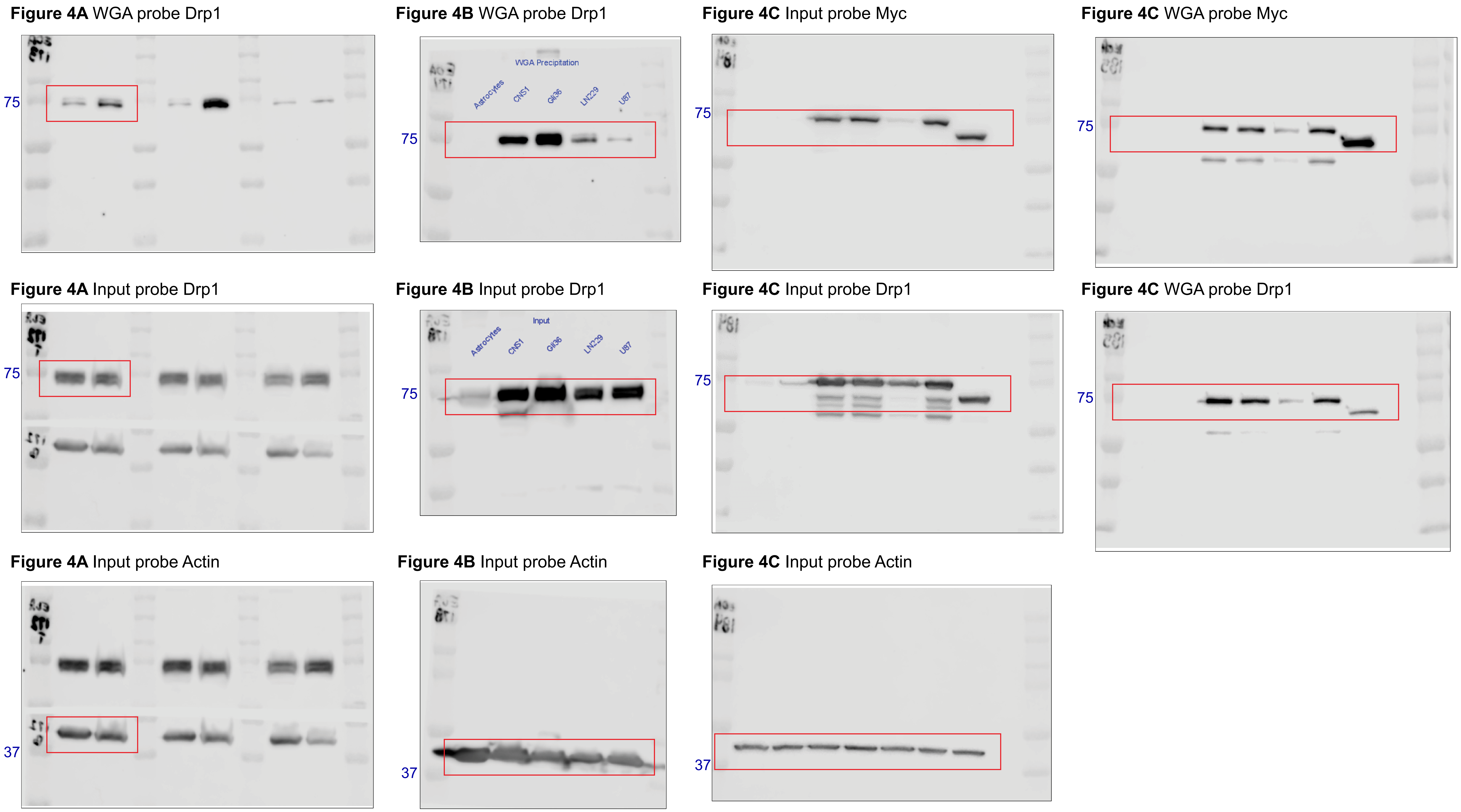

Supplement: Supplementary file 1 — Supplementary Information. [file 41598_2021_1512_MOESM1_ESM.pdf]
